# Diagnostic Imaging of Canine Hepatobiliary Affections: A Review

**DOI:** 10.1155/2012/672107

**Published:** 2012-03-18

**Authors:** Vijay Kumar, Adarsh Kumar, A. C. Varshney, S. P. Tyagi, M. S. Kanwar, S. K. Sharma

**Affiliations:** Department of Veterinary Surgery and Radiology, DGCN College of Veterinary and Animal Sciences, CSK HP Agricultural University, Himachal, Palampur Pradesh 176062, India

## Abstract

Hepatic disease is often treatable and has a predictable prognosis when a definitive diagnosis is made. The aim of clinicopathological evaluation of hepatobiliary affections is to identify and characterize hepatic damage and dysfunction, identify possible primary causes of secondary liver disease, differentiate causes of icterus, evaluate potential anaesthetic risks, assess prognosis and response to xenobiotics, and monitor response to therapy. This paper describes the different diagnostic methods and imaging techniques employed in diagnosis of hepatobiliary affections in dogs. Besides reviewing the significant clinical manifestations and imaging structural abnormalities in diagnostic approach to different hepatic affections, it also depicts radiographic, ultrasonographic, and wherever applicable, the laparoscopic characterization of different hepatic affections and target lesions encountered in clinical cases presented in the Teaching Veterinary Clinical Complex, COVAS, Palampur in the year 2007-2008.

## 1. Introduction

Hepatic affections in the dog are associated with varied and often vague clinical signs and thus frequently present a diagnostic challenge to veterinary practitioners. As the liver has great functional reserve capacity, detection of the hepatic functional impairment by conventional means is only possible once a significant hepatic dysfunction (≥55%) is present [1]. Furthermore, systemic diseases and various drugs can cause misleading increases in serum activities (secondary or reactive hepatopathies), and it can be a clinical dilemma to decide whether liver enzyme elevations are significant, and whether they represent primary or secondary liver disease [2]. Despite availability of a range of diagnostic tests of both hepatic damage and dysfunction, there is rarely a single test that adequately identifies hepatic disease or its underlying cause.

The role of survey radiography in detecting the alteration in liver size has been widely emphasized. But with the advent of time, ultrasonography evolved as the paramount technique of diagnostic imaging. The desirable virtues of less time consumption and noninvasive assessment of the detailed internal architecture of the liver and the adjacent structures, including the portal vein, established ultrasonography as the choicest technology, for identifying the various forms of hepatic disease in canines [2]. Presently, laparoscopy is also emerging as a fruitful imaging modality. It offers tremendous advantage of direct visceral visual inspection of liver and allows its descriptive or photographic documentation. This technique provides three-dimensional evaluations of liver and is also a minimal invasive method to obtain cytology, biopsy, and cultural samples from focal lesions or generalized disease condition/carcinomatous growth of liver [3].

It is important to interpret all results in light of the other aspects of the diagnostic investigation, in particular the history and physical examination. In most cases, a tentative diagnosis of primary hepatic disease can be deduced by correlating the ultrasonographic abnormalities with the history, physical examination findings, clinical laboratory results, and radiographic/laparoscopic observations. However, for identification of specific hepatopathies and thus establishment of definitive diagnosis of primary liver disease, the histopathological examination of the liver biopsy specimens is usually required.

## 2. Diagnostic Tests and Imaging Techniques for the Hepatobiliary Affections

By using a combination of history, physical examination findings, results of screening, and hepatobiliary-specific laboratory tests, the clinician usually becomes apt to describe the disorder as active or quiescent. Also, it becomes easy for him/her to characterize the pattern of hepatobiliary disease (primarily hepatocellular, primary biliary, or mixed hepatobiliary) and further estimate the degree of hepatobiliary dysfunction [2]. So in the light of all these aspects, a complete evaluation comprising of the following tests, must be conducted.

### 2.1. Laboratory Evaluation Tests

#### 2.1.1. Complete Blood Count

The complete blood count (CBC) is an integral part of the diagnostic investigation of any systemic disease process or for that matter of hepatic affections. It consists of quantitative and qualitative examination. The quantitative examination includes packed cell volume (PCV), total red blood cell count (RBC), haemoglobin (Hb) concentration, total white blood cell count (WBC), differential WBC count and platelet count. In addition, the red cell mean corpuscular volume (MCV), mean corpuscular haemoglobin (MCH), and mean corpuscular haemoglobin concentration (MCHC) are also evaluated. The qualitative examination includes examination of blood smears for changes in cellular morphology.

#### 2.1.2. Serum Biochemistry

Biochemical findings often prove to be the most useful aid in the diagnosis of hepatobiliary affections. Liver-specific serum enzyme activities are included routinely in screening serum biochemistry panels and are regarded as markers of hepatocellular and biliary injury and reactivity [2]. In addition to this, many biochemical tests are not specific indicators of liver disease, but do offer a crude assessment of liver status, or aid recognition of diseases that either mimic the clinical signs of liver disease or actually cause secondary liver disease. The minimum serum biochemical database comprises of ALT, AST, ALP, GGT, BUN, creatinine, total bilirubin, total protein, glucose, albumin, globulin, and cholesterol in suspected hepatic affections [1].

#### 2.1.3. Urinalysis and Faecal Analysis

Urine analysis provides rapid and valuable information about the urinary tract and other body systems including liver. A complete urine analysis (including dipstick, specific gravity (SG), and sediment examination) is often required, even if one component part shows no abnormalities [2]. Concurrent serum biochemical analysis, although, is often required to gain maximum benefit from urine analysis. There is definite change in the appearance of the faeces in hepatobiliary affections. So, the faecal specimen analysis must be conducted although it rarely provides useful information in the evaluation of the dog with suspected hepatobiliary affection.

#### 2.1.4. Abdominocentesis

**Figure 1:**
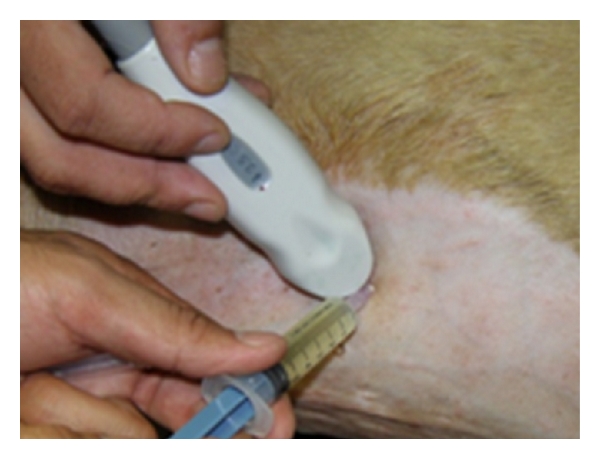
Six-year-old neutered male Labrador Retriever dog positioned in left lateral recumbency for ultrasound-guided abdominocentesis. The needle is directed perpendicular to the abdominal wall, with care taken to avoid the spleen.

**Figure 2:**
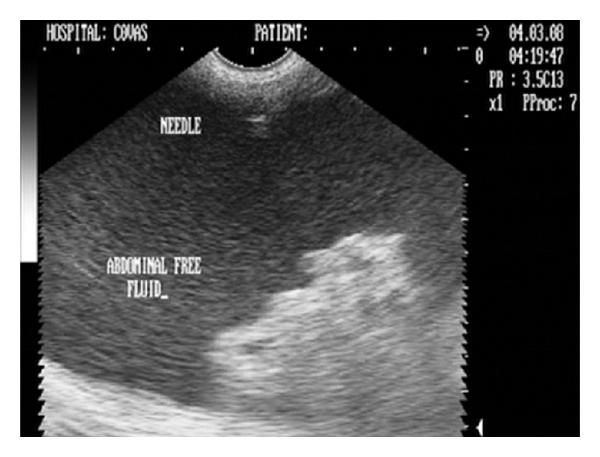
Two-dimensional ultrasonographic appearance of 22-gauge needle as hyperechoic structure within the textured abdominal effusion in 6-year-old male Labrador Retriever affected with infectious peritonitis.

**Figure 3:**
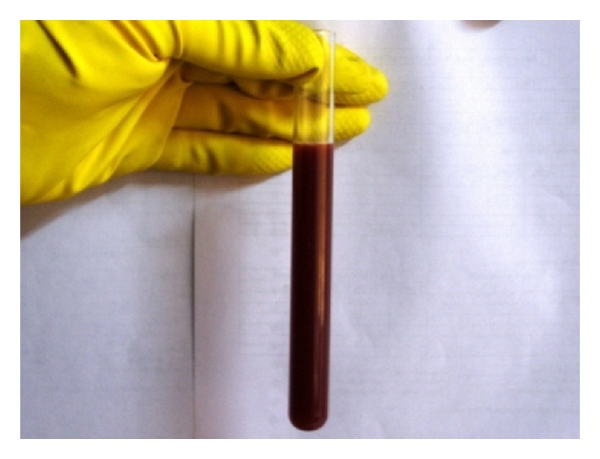
Gross appearance of the abdominal effusion (septic exudate) obtained under ultrasound guidance from 6-year-old male Labrador Retriever affected with infectious peritonitis.

Analysis of abdominal effusions is an important component of diagnosis. It can either assist in timely identifying the pathological process responsible for the fluid accumulation or it can help in indicating further investigative procedures which may be helpful in diagnosing the affection [4]. Abdominocentesis can be performed with the patient standing or in left lateral recumbency either with blind percutaneous abdominocentesis or with ultrasound-guided abdominocentesis (Figures 1 and 2). The abdominal effusion so obtained is analysed by subjecting it to gross examination, cytological examination, and microbiological examination. Besides, total protein concentration and total nucleated cell count is also evaluated [2]. The effusion analysed is then classified as transudate, modified transudate, or exudates (Figure 3).

#### 2.1.5. Coagulation Tests

The liver plays a central role in the coagulation and fibrinolytic systems, and subtle abnormalities may be detected by assay of individual factor activities. Whilst a bleeding diathesis will be expected if there is a history of gastrointestinal bleeding, an occult tendency should always be suspected, and a clotting profile is mandatory before a liver biopsy is performed [3]. The coagulation profile should comprise evaluation of buccal mucosal bleeding time, whole blood clotting time, one-stage prothrombin time (OSPT or PT), and activated partial thromboplastin time (aPTT). In conjunction to it, an assessment of fibrin degradation products (FDPs), D-dimers, and vitamin-K should also be made [2].

#### 2.1.6. Dynamic Liver Function Tests

These tests can be very useful in diagnosing hepatic affections, with the exception of those patients, who are icteric. These tests rely on analysis of paired blood samples to assess the capacity of the liver to clear endogenous (bile acids and ammonia) or exogenous (bromosulphthalein and indocyanine green) substances from the circulation [2]. Impaired clearance although suggests hepatocellular dysfunction and/or portosystemic shunting, but does not differentiate the cause. Therefore, additional tests, including portovenography, ultrasonography, and liver biopsy are required [2].

### 2.2. Radiography

**Figure 4:**
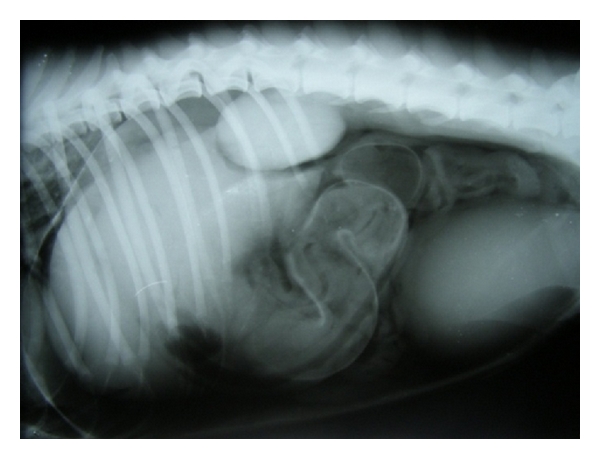
Left lateral capnoperitoneograph enhancing visualization of right liver lobes and diaphragmatic border in 8-year-old male Dobermann Pinscher dog demonstrating classical signs of pulmonary metastasis with strong suspicion for hepatic neoplasia.

**Figure 5:**
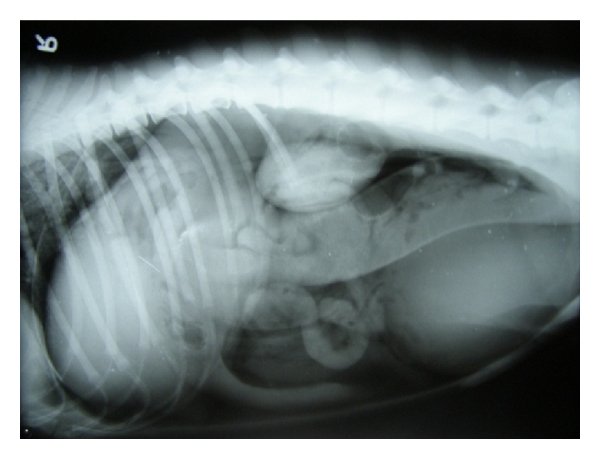
Right lateral capnoperitoneograph enhancing visualization of left liver lobes and diaphragmatic border in 8-year-old male Dobermann Pinscher dog demonstrating classical signs of pulmonary metastasis with strong suspicion for hepatic neoplasia.

Survey abdominal radiographs (lateral and ventrodorsal view) are useful to evaluate the morphologic abnormalities in size, shape, position, and density (mineralization/radiolucencies) of the liver and presence of abdominal effusion. However, lack of abdominal contrast and insensitivity to detect subtle changes limits the precision of abdominal radiography. It is difficult to evaluate the entire liver as much of the liver is silhouetted by the diaphragm, stomach and right kidney [5]. Capnoperitoneography, the special contrast radiographic procedure, enhances the visceral visualization of abdominal organs in general and is very useful in the evaluation of liver lobes and its borders, especially the diaphragmatic border [6, 7] (Figures 4 and 5). In suspected cases of hepatic neoplasia, thoracic films to evaluate the pulmonary metastasis are also desired.

**Figure 6:**
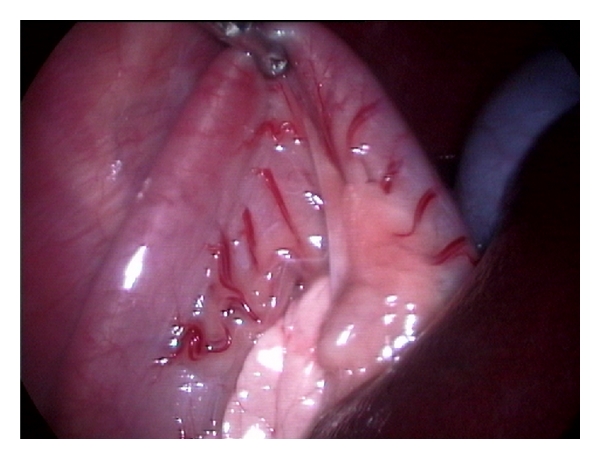
Grasping of jejunal loop under laparoscopic-guidance for performing laparoscopic guided and laparotomy assisted introperative mesenteric portography in 1-year-old male mixed breed dog with progressive weakness.

**Figure 7:**
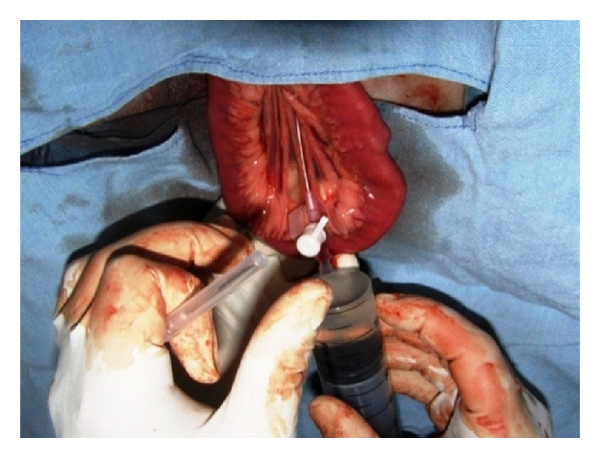
Laparotomy-assisted exteriorization of jejunal loop and catheterization of jejunal vein and rapid infusion of positive contrast agent (Urografin 76%) in 1-year-old male mixed-breed dog, suspected for portosystemic shunting.

**Figure 8:**
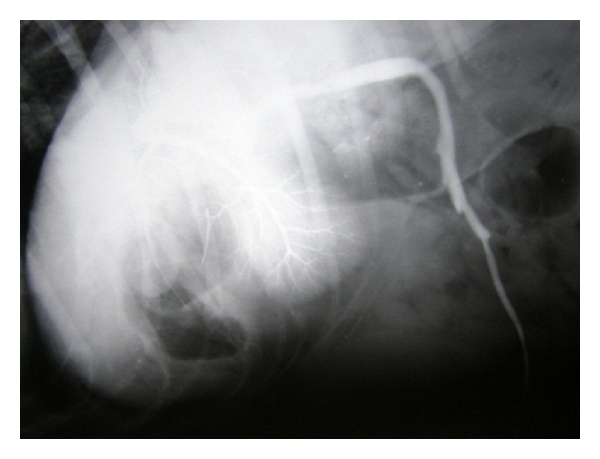
Normal-appearing hepatic angiogram, demonstrating jejunal mesenteric vein normally draining into the portal vein and the extensive portal vein branches in1-year-old male mixed-breed dog.

**Figure 9:**
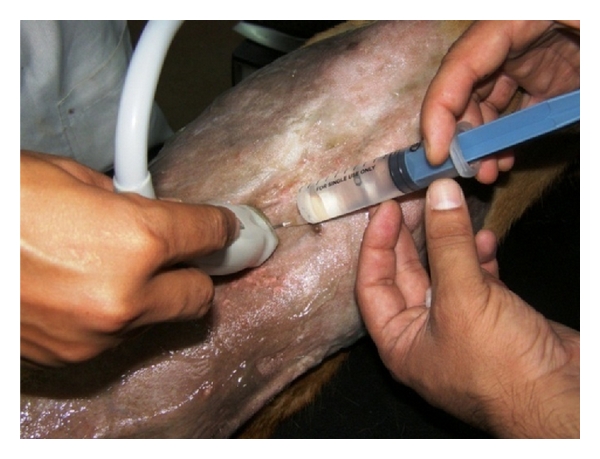
Ultrasound-guided percutaneous injection of positive contrast agent (Urografin 76%) in 3-year-old male Labrador Retriever dog, suspected for portosystemic shunting.

**Figure 10:**
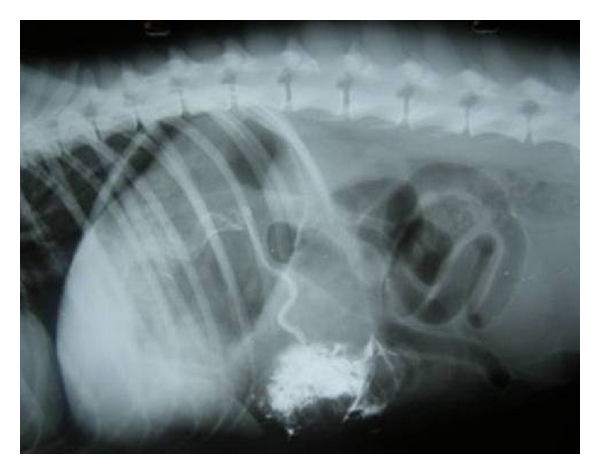
Ultrasound-guided percutaneous normal-appearing splenic portogram demonstrating splenic vein normally draining into portal vein with hepatic contrast opacification in 3-year-old male Labrador Retriever dog.

In addition to these, laparoscopic-guided intraoperative mesenteric portography (Figures 6, 7, and 8) and/or ultrasound-guided percutaneous splenoportography (Figures 9 and 10) are easily executable procedures to evaluate the hepatic blood flow in clinical settings.

### 2.3. Ultrasonography

Ultrasonography is an excellent noninvasive way to evaluate liver parenchyma. It is particularly useful in differentiating focal from diffuse disease, cystic from solid masses and obstructive from nonobstructive icterus [5]. Indications for hepatic ultrasound usually include elevated liver enzymes and presence of free abdominal effusion. This procedure is also indicated for determining the extent of abdominal metastasis in cases of hepatic neoplasia and to image congenital or acquired portocaval or portosystemic shunts. Doppler imaging confirms the location of the suspicious vessels and direction of the blood flow within and can also provide supportive evidence of intrahepatic portal hypertension by allowing the assessment of the speed and direction of portal flow [2]. Besides, ultrasound-guided percutaneous hepatic biopsy allows precise direction of the biopsy needle to the area of abdominal tissue while avoiding large vessels, the gall bladder, and the gastrointestinal tract [5].

### 2.4. Laparoscopy

Laparoscopy offers tremendous advantage of direct visceral visualization (three-dimensional) of the liver and adjacent structures such as the pancreas and extrahepatic biliary tract. Laparoscopy may reveal very small (0.5 cm or less) metastatic lesions that are not easily observed by other diagnostic techniques. Laparoscopy may also provide accurate, definitive and staging information that otherwise would have been obtained only through a surgical laparotomy. It also provides minimal invasive method to obtain cytology, biopsy, and cultural samples from focal hepatic lesions or generalized disease condition/carcinomatous growth [3]. It is seen that laparoscopy provides better liver biopsy tissues than any other traditional percutaneous methods especially when the liver is small [1]. It also provides the advantage of procuring biopsy from areas visually that are less vascular and to monitor the extent of bleeding after a biopsy [3]. It is preferred to percutaneous techniques when excess bleeding is expected and to laparotomy when delayed wound healing (hypoalbuminaemia) is anticipated. The minimal invasiveness of the procedure, rapid patient recovery, and diagnostic accuracy make laparoscopy an ideal technique compared with more invasive procedures. Despite the advent of newer laboratory tests, imaging techniques, and ultrasound-directed fine-needle biopsy or aspiration, laparoscopy remains a valuable tool when appropriately applied in a diagnostic plan. Ascites, abnormal clotting times, small body size (<2 kg of body weight), and poor patient condition are the only relative contraindications to laparoscopy. This technique requires heavy sedation or anaesthesia and is subject to equipment availability and clinician expertise.

### 2.5. Scintigraphy, Magnetic Resonance Imaging (MRI), and Computed Tomography

Scintigraphy (nuclear imaging), magnetic resonance imaging (MRI), and computed tomography have recently been used in the diagnosis of hepatobiliary affections, but the need for radioisotopes and expensive equipment has restricted their use to the teaching institutions of developed nations. Of these three imaging modalities, scintigraphy has been thoroughly evaluated for diagnosis of hepatobiliary affections in canines [2]. It employs the use of isotope technetium 99 m (^99m^Tc), which is incorporated into the radiopharmaceutical specific for the planned study. After an intravenous injection of radiopharmaceutical, scintigraphic images are made sequentially over 3 hours to determine whether isotope has been taken up by the liver, excreted into the biliary tract, and expelled into the intestine. In canines with extrahepatic bile duct obstruction, no evidence of radiopharmaceutical is detected in the gall bladder or intestine. Another application of scintigraphy is used in the diagnosis of PSS in canines.

### 2.6. Liver Biopsy and Histopathological Examination

**Figure 11:**
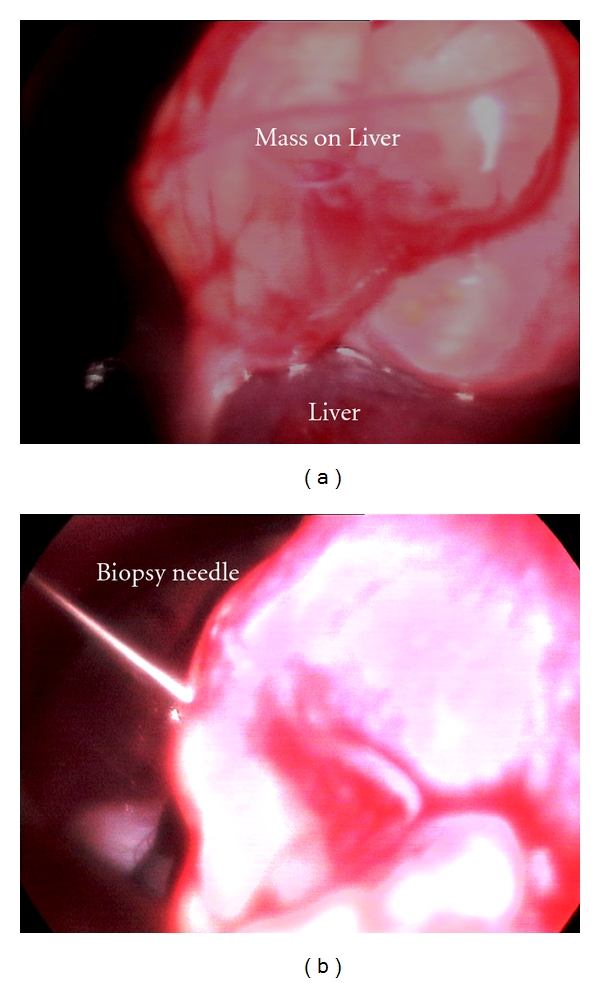
Laparoscopic-guided percutaneous Tru-Cut needle biopsy of hepatic mass in 8-year-old intact male Labrador Retriever dog.

**Figure 12:**
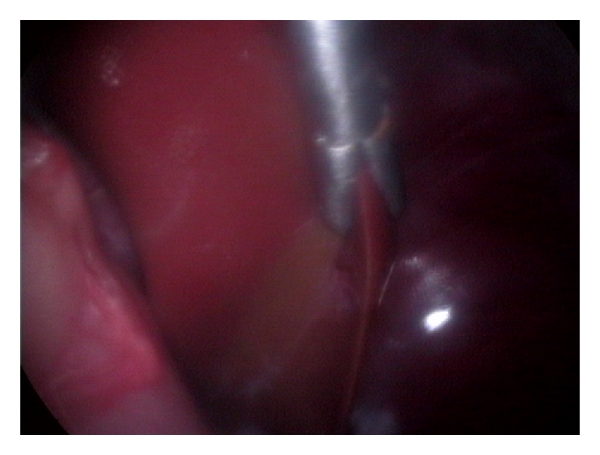
Laparoscopic procurement of liver biopsy (right flank approach) depicting oval biopsy forceps grasping the hepatic nodule at the edge of right medial liver lobe in 7-year-old male mixed-breed dog with hepatic nodular hyperplasia and radiographic appreciation of pulmonary metastasis.

**Figure 13:**
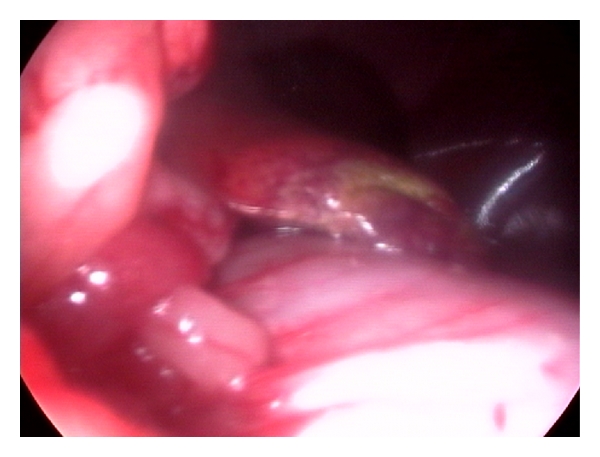
Laparoscopic postprocurement monitoring of liver biopsy site for bleeding in 7-year-old male mixed-breed dog with hepatic nodular hyperplasia.

**Figure 14:**
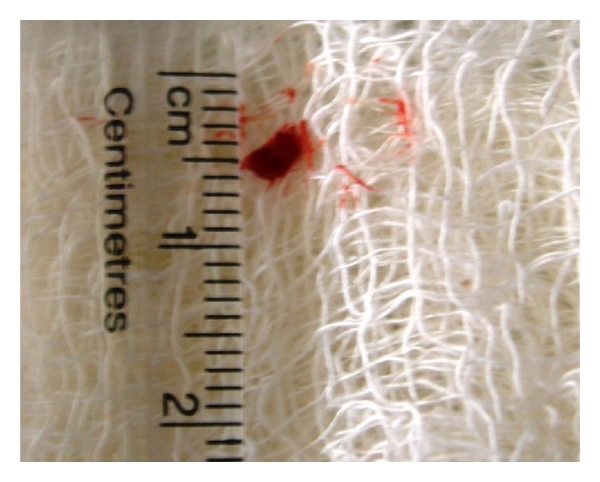
Laparoscopic Procurement of 3 mm liver tissue in 7-year-old male mixed-breed dog with hepatic nodular hyperplasia.

Liver biopsy is often required to definitely characterize the nature and severity of the hepatic disease. It can be further used to differentiate acute from chronic disorders, to stage neoplastic disease and to assess response to therapy. Selection of the best procedure for obtaining a liver biopsy depends on numerous factors including liver size, presence of coagulopathy, any focal or diffuse lesion, presence of biliary tract obstruction, or any other intra-abdominal abnormalities. The selection of the biopsy method also depends on likelihood of surgical resection of a mass, tolerance of general anaesthesia, available equipment and expertise of the clinician [2]. The various biopsy methods include fine-needle aspiration, blind percutaneous needle biopsy using Tru-Cut biopsy needle, ultrasound-guided needle biopsy, keyhole needle biopsy, and laparoscopic-guided biopsy [8] (Figures 11, 12, 13, and 14). The biopsy specimens so procured are subjected to standardized processing and histopathological examination for yielding definitive diagnosis of hepatic affections.

## 3. Diagnostic Features/Alterations in Different Hepatobiliary Affections

The following canine hepatopathies have been reviewed according to their prevalence in clinical practice.

### 3.1. Chronic Hepatitis

#### 3.1.1. Etiology

In idiopathic chronic hepatitis, it is probable that after an initial inciting hepatocyte injury, immune mechanisms are involved in perpetuating the inflammation [8]. Familial chronic hepatitis in Bedlington terriers, Dobermann pinschers, West Highland White Terriers and Skye Terriers have a hereditary (autosomal recessive) inability to excrete copper in the bile that is associated with progressive hepatic copper accumulation and chronic liver disease [9]. Chronic drug administration (heartworm preventatives, anticonvulsants, glucocorticoids, and chemotherapeutic drugs) is a frequent cause of chronic hepatitis in canines [8]. Latent hepatic viral infection with canine adenovirus type I, which is responsible for infectious canine hepatitis (ICH), is also suspected to cause persistent inflammation and thus chronic hepatitis [9]. Cirrhosis is the irreversible end stage of the chronic hepatic injury caused by the infection, hepatotoxins (copper and anticonvulsants), immunologic injury (chronic hepatitis), chronic cholestasis, and hypoxia [9]. Hepatic inflammation, regeneration and fibrosis result in the development of portal hypertension and the establishment of multiple extrahepatic portosystemic shunts which in turn promote the development of ascites [1, 8], whereas perihepatic changes such as ascites, portal and splenic vein dilation and splenomegaly might occur with cirrhosis because of portal hypertension [10].

#### 3.1.2. Clinical Manifestations

**Figure 15:**
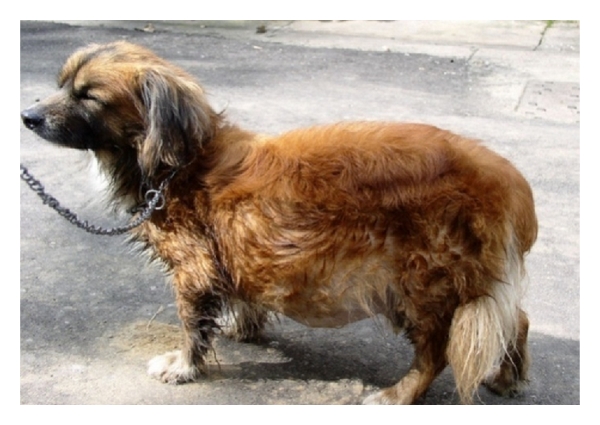
4-year-old male mixed-breed dog with chronic hepatitis manifesting severe abdominal enlargement (ascites) during physical examination.

Dogs affected by this condition are mostly young or middle aged adults of either sex with signs of hepatitis of varying severity. The symptoms included lethargy, depression, weight loss, vomiting, and jaundice. Idiopathic chronic hepatitis, however, appears to be highest in female dogs with signs of anorexia, depression, weakness, polyuria/polydipsia, ascites, jaundice, weight loss, and vomiting [9]. The physical examination findings with copper-associated acute hepatitis may include depression, lethargy, dehydration, jaundice, and hepatomegaly. With end-stage hepatic disease, the signs included dehydration, emaciation/muscle wasting, ascites, and abnormal mentation (hepatic encephalopathy) with jaundice and a nonpalpable small liver [9] (Figure 15).

#### 3.1.3. Laboratory Evaluation

Erythrocytic morphologic changes which include variable red cell shapes (poikilocytes) with irregularly speculated erythrocytes (acanthocytes or spur cells) and target cells are considered to be consistent with chronic hepatobiliary disease [9]. An inflammatory leucogram is also sometimes seen in severe chronic inflammatory hepatopathies [8]. Consistent serum chemistry findings include increased ALT and ALP activity reflecting ongoing hepatic injury and intrahepatic cholestasis respectively, whereas less consistent findings include hypoalbuminaemia, hyperglobulinaemia, and abnormal haemostasis [9]. Ascitic fluid, when present, typically is a transudate or modified transudate [1].

#### 3.1.4. Radiographic Features

**Figure 16:**
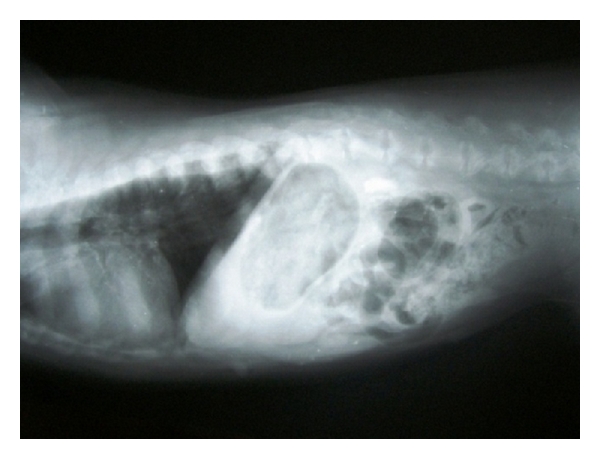
Right lateral radiograph of a 2-year-old castrated male mixed-breed dog with microhepatica. The stomach is distended with gas, which provides contrast with the liver shadow. Notice the proximity of the stomach to the diaphragm, implying reduced hepatic mass.

**Figure 17:**
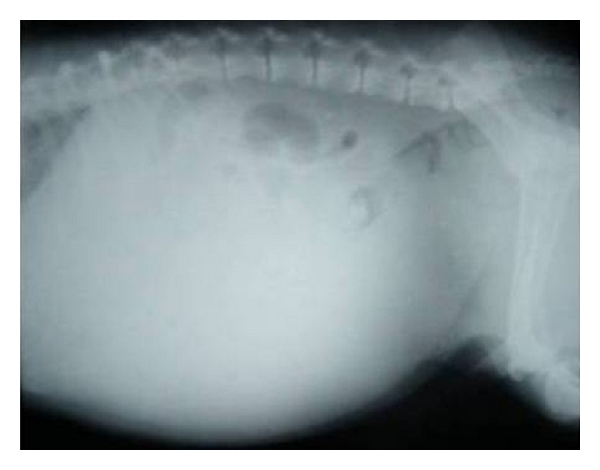
Lateral standing abdominal radiograph of a 7-year-old male mixed-breed ascitic dog with classic “ground glass appearance” of abdomen and masking of abdominal cavity details.

Radiographic appearance of cirrhosed liver varies with the stage and severity of the disease and a small, dense liver with an irregular nodular surface may be identified (with or without pneumoperitoneograph) most commonly in cirrhosis [7]. However, radiographic appreciation of reduced liver size (microhepatica) is more difficult than hepatomegaly [11] (Figure 16). The abdominal effusion in ascitic dogs precludes the radiographic examination of the liver and other abdominal organs with its classic “ground glass” appearance (Figure 17).

#### 3.1.5. Ultrasonographic Features

**Figure 18:**
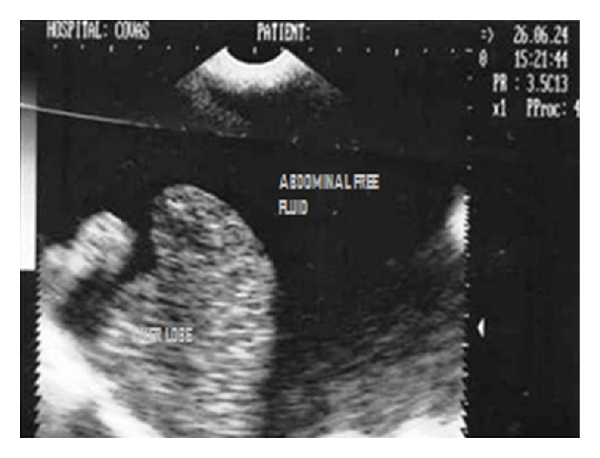
Ultrasonograph (2D) in sagittal scan of shrunk liver with hyperechoic hepatic parenchyma and irregular lobe margins surrounded with anechoic abdominal effusion in a 5-year-old intact male Cocker spaniel dog.

**Figure 19:**
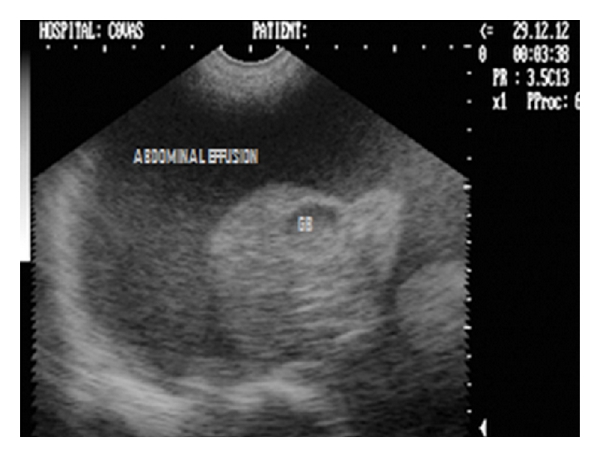
Sonograph (2D) in transverse scan showing hyperechoic liver lobe and cholecystitis with inspissated bile in gall bladder (GB) in a 7-year-old intact Pomeranian male dog.

Sonographic features in cases of cirrhosis vary from multiple hepatic nodules (from macronodular regeneration) besides hyperechoic hepatic parenchyma and decreased liver lobe size [12] to diffuse hyperechoic (bright) but small liver with distended gall bladder and irregular contour [13, 14]. Cirrhosis can be visualized sonographically as increased hepatic echogenicity accompanied with less distinct appearance of the echogenic portal vein margins and decreased distal visualization. This specific ultrasonographic finding is because of increased beam attenuation. Periportal fibrosis in cirrhotic liver is suggested by abnormally heterogenous hepatic echogenicity giving it a mottled appearance and abnormally prominent marginal echoes of the intrahepatic branches of the portal vein [15]. Increase in hepatic echogenicity may be present in disease conditions like cirrhosis, hepatic lipidosis, steroid hepatopathy, lymphosarcoma, long-term cholangiohepatitis, and some toxic hepatopathies [16, 17] (Figures 18 and 19).

#### 3.1.6. Histopathological Features

The primary lesion is portal inflammation consisting primarily of lymphocytes and plasma cells; occasional neutrophils and macrophages [8]. With idiopathic chronic hepatitis, the inflammation extends into the hepatic lobule, causing piecemeal necrosis of hepatocytes [9]. In familial chronic hepatitis, H and E-stained hepatic tissue reveals dark granules in hepatocyte cytoplasm in centrilobular hepatocytes in the early stages and generalized in later stages [9]. Microscopic features include fibrosis, regenerative nodules, and disruption of normal hepatic architecture.

### 3.2. Noncirrhotic Portal Hypertension

Three unusual diseases of primarily young dogs, that is, hepatoportal fibrosis, idiopathic hepatic fibrosis, and primary hypoplasia of portal vein might be grouped under this title.

#### 3.2.1. Etiology

Portal hypertension can have prehepatic causes such as portal vein thrombosis, stenosis, or compression by enlarged portal lymph nodes/neoplastic masses. Posthepatic causes include compression of hepatic veins, caudal vena cava, or right heart disease [18]. Portal hypertension may be secondary manifestation of right-sided congestive heart failure, caudal vena cava obstruction, and intrahepatic obstruction [19].

#### 3.2.2. Clinical Manifestations

Most affected dogs are presented as young or middle aged dogs (≤2.5 years) of either sex with signs of hepatic failure including lethargy, depression, weight loss, jaundice, and ascites [9]. Abnormal mentation due to hepatic encephalopathy may be a less consistent sign of this disorder [8].

#### 3.2.3. Laboratory Evaluation

Microcytosis is a common feature with this disorder. Noncirrhotic portal hypertension disorders are characterized paradoxically by abnormal liver enzyme activities and hyperbilirubinemia in about 50% of the cases [9].

#### 3.2.4. Radiographic Features

The abdominal effusion precludes the radiographic examination of the liver and other abdominal organs as ascites is always a feature with noncirrhotic hypertension [9].

#### 3.2.5. Sonographic Features

**Figure 20:**
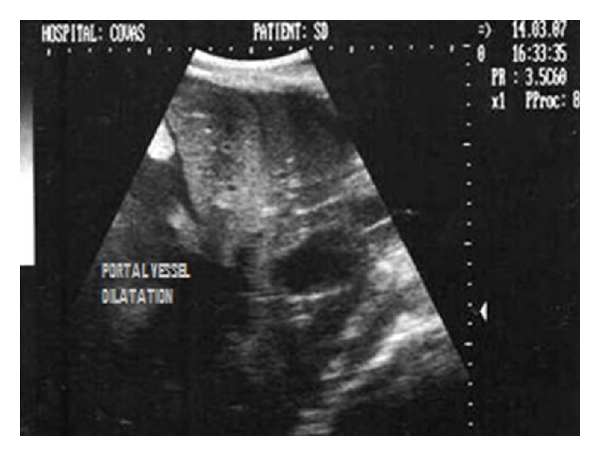
Dorsal sonogram (2D) of liver depicting hypoechoic parenchyma with portal vessel dilatation in one-year-old mixed-breed dog.

**Figure 21:**
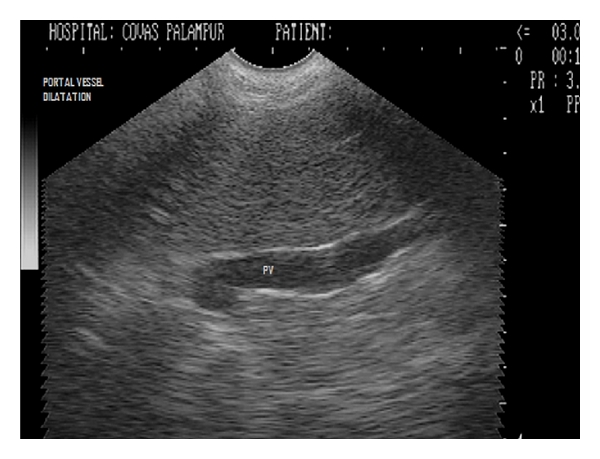
Longitudinal sonogram (2D) of liver depicting hypoechoic parenchyma with marked portal vessel dilatation (subjective) in 5-year-old mixed-breed dog with significant spleenomegaly.

Portal hypertension is difficult to diagnose with standard ultrasound imaging. Besides the presence of multiple portosystemic collateral vessels, ascites, splenomegaly, and an abnormal liver echogenicity, enlarged main portal and extrahepatic portal veins are the associated features with portal hypertension [20]. Ultrasonography is an effective tool for evaluation of the presence of portal hypertension and assessment of its effects [18] (Figures 20 and 21).

#### 3.2.6. Histopathological Features

The liver biopsy findings and histopathological examination may range from unremarkable to minimal inflammatory changes in disorders of non-cirrhotic portal hypertension [9].

### 3.3. Congenital Portovascular Anomalies

#### 3.3.1. Etiology

The most common patterns of portovascular anomaly are single-extrahepatic communications between the portal vein or one of the mesenteric veins and the caudal vena cava or azygos vein in small-breed dogs and patent ductus venosus in large-breed dogs [9]. Hypoplasia or aplasia of intrahepatic portal vasculature could complicate any of these anomalies, but it is rare.

#### 3.3.2. Clinical Manifestations

Age range of affected dogs of either sex is 2 months to 8 years; most are presented when less than 1 year old. The common clinical signs in canines with single intrahepatic or extrahepatic PSS are those of hepatic encephalopathy not always related to meal ingestion and/or gastrointestinal disturbance such as vomiting, diarrhea, or pica [9]. The presenting complaints are polyuria and polydipsia, urate urolithiasis, and anaesthetic or sedative intolerance. Physical examination findings include small size, poor haircoat, and occasional renal enlargement [8]. Ascitis is seen in canines with intrahepatic arteriovenous fistula. The clinical findings in uncomplicated congenital or acquired portosystemic shunts are unremarkable except for small body size or weight loss whereas ascites and continuous murmur over the area of the liver is a consistent finding with hepatic arteriovenous fistulas/secondary portosystemic shunts [9].

#### 3.3.3. Laboratory Evaluation

Clinicopathologic findings in over 50% of the affected dogs, regardless of the type of vascular anomaly, are microcytosis, hypoalbuminaemia, mild increases in serum ALT and ALP activities, hypocholesterolemia, low-BUN content, postchallenge hyperammonemia, and normal or high fasting with high postprandial SBA [9].

#### 3.3.4. Radiographic Features

A small-sized liver is a frequent radiographic appreciation with any type of vascular anomaly [8]. To confirm the location of the anomalous vessel, combinations of ultrasonography, transcolonic scintigraphy, or contrast portal venography may be needed [9].

#### 3.3.5. Ultrasonographic Features

Routine abdominal ultrasonography in canines with portovascular anomalies may demonstrate intrahepatic and extrahepatic shunts with reduced hepatic volume and urinary calculi [8]. Markedly increased and abnormally variable portal blood flow velocity with attenuated intrahepatic portal vessels and localized dilatation of the intrahepatic portal vein (PV) at the site of communication with the caudal vena cava (CVC) may be detected in congenital intrahepatic portosystemic shunts. Whereas, in congenital extrahepatic portozygos shunt, the shunting vessel runs roughly parallel to and on the left of the abdominal caudal vena cava and could be followed to the diaphragm without communicating with the caudal vena cava [21].

#### 3.3.6. Histopathological Features

Liver biopsy most consistently reveals hepatocyte atrophy with small or absent portal veins. Varying degrees of sinusoidal congestion, biliary hyperplasia, arteriolar proliferation, lipogranulomata, and increased periportal connective tissue may be seen [8, 9].

### 3.4. Acute Hepatic Failure

#### 3.4.1. Etiology

Causes of acute hepatic failure in canines include hepatotoxins, infectious and parasitic agents, and miscellaneous disorders [8]. Certain environmental toxins (pesticides, herbicides, cleaning agents and plant toxins), have inherent ability to cause hepatic injury either by direct hepatocellular damage or by disturbance of hepatocellular homeostasis, which results in cell death [9]. In addition to this, certain antimicrobial drugs (ketoconazole and trimethoprim-sulfa), antihelminths (mebendazole, diethylcarbamazine-oxybendazole, and thiacetarsamide), inhalation anesthetics (halothane and methoxyflurane), and analgesics (acetaminophen, naproxen, phenylbutazone) have also been reported to be hepatotoxic in canines [8, 9]. The various infectious causes of acute hepatic failure include leptospirosis, toxoplasmosis, histoplasmosis, and infectious canine hepatitis. Besides, other systemic conditions like immune-mediated haemolytic anaemia, surgical hypotension, hypoxia, shock, acute pancreatitis, and extrahepatic bacterial infections (pneumonia, pyometra, peritonitis, and abscesses) can also cause severe hepatic injury [8].

#### 3.4.2. Clinical Manifestations

Nutritional status and gender are important host factors with females being more susceptible to toxic liver injury than males [9]. Dose, duration of exposure, and chemical composition of the agent contribute to how toxicity is expressed by the host. The clinical signs and clinicopathologic features of acute toxic hepatopathy are not distinct from other hepatopathies except in onset and perhaps in severity. Vomiting is a consistent sign with acute toxic hepatopathy. Hepatodynia is a feature with any cause of acute hepatic injury, whereas fever and acute abdominal pain are presenting signs of acute pancreatitis, cholangiohepatitis, and hepatic abscess [8, 9]. Fever may be present with infectious causes of hepatic injury such as leptospirosis, ICH, bacterial cholangiohepatitis, liver abscess, systemic mycoses, and extrahepatic infections [8].

#### 3.4.3. Laboratory Evaluation

An inflammatory CBC in acute hepatic failure may suggest acute pancreatitis or underlying infectious disease. The clinicopathologic test results are typical of mild, moderate, or severe hepatocellular damage, suggested by high-serum ALT activity and variable increases in ALP activity [8]. Other potential findings include hyperbilirubinemia, increased SBA concentration, hypoglycaemia, hyperammonemia, and coagulopathy. Biochemical evidence of concomitant renal failure may be present in acute hepatic failure as sequel to exposure to hepatotoxins (e.g., thiacetarsamide and inhalation anesthetics) and infectious agents (e.g., leptospirosis) [9].

#### 3.4.4. Radiographic Features

Radiographic examination may be unremarkable or may reveal hepatomegaly. Massive hepatic necrosis causing hepatic parenchymal collapse and radiographic appreciation of microhepatica although possible is less consistent with acute hepatic injury [9].

#### 3.4.5. Ultrasonographic Features

**Figure 22:**
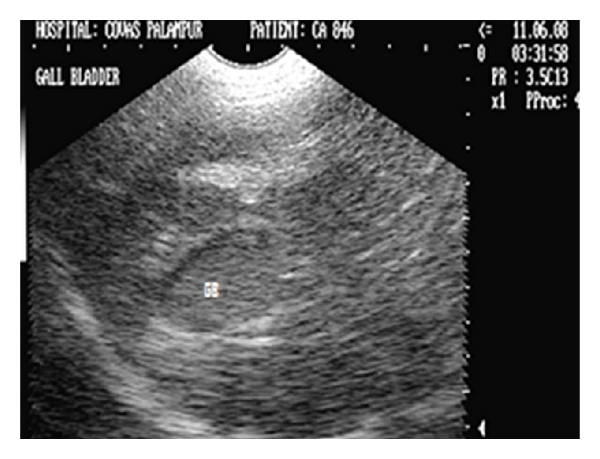
Transverse sonogram (2D) of right medial liver lobe of two and half-year-old intact female Pointer dog depicting generalized hyperechoic parenchyma with biliary sludge in gall bladder.

**Figure 23:**
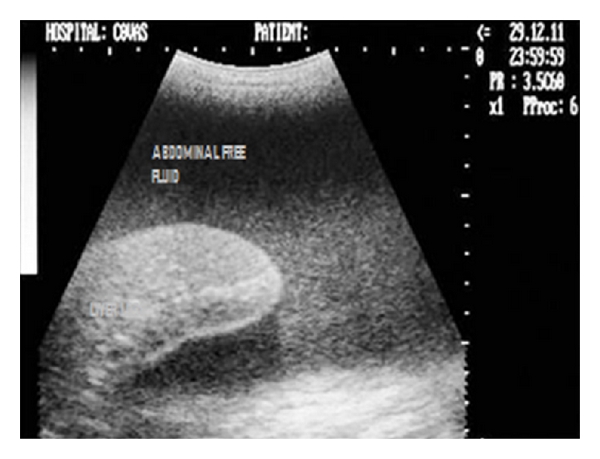
Two-dimensional ultrasonographic appearance of liver in sagittal scan depicting hyperechoic parenchyma with rounding of liver lobe surrounded with textured fluid in a 6-year-old male Labrador Retriever affected with infectious peritonitis.

Acute hepatic failure may be characterized by hepatomegaly with normal, increased or decreased parenchymal echogenicity along with diffuse parenchymal abnormalities wherein the discrete hepatic borders are not visualized [22]. The hepatic parenchymal echogenicity in canines with acute hepatic failure may range from normal to diffusely mottled to decreased echogenicity [23]. Passive congestion observed in acute hepatic insult is the most common benign cause of diffuse decreased liver echogenicity which result from dilatation of hepatic sinusoids and hepatitis, owing to swelling of hepatocytes [24] (Figure 22). Symmetrical changes in hepatic volume in hepatomegaly can be estimated by measuring the maximal distance from the caudal tip of the liver on the ventral midline till the diaphragm. On a sagittal image, there is an approximately linear relationship between this dimension and body weight [25]. Large liver lobes in hepatomegaly in acute hepatic insults often have rounded margins, whereas the normal liver lobes have sharp edges [26]. The diffuse hypoechoic hepatic parenchyma and rounded liver margins with hepatomegaly have been documented in acute hepatic failure as varied sonographic findings ranging from normal to diffusely mottled or decreased echogenicity [23, 26] (Figure 23).

#### 3.4.6. Histopathological Features

Diffuse hepatic necrosis is the primary histologic lesion most consistently associated with acute hepatic failure [8]. Histopathologic lesions described thus far in acute-to-subacute drug-induced hepatopathy in canines are centrilobular necrosis or periportal inflammation, either of which can be features of other hepatopathies [9].

### 3.5. Biliary Tract Disorders

Disorders of biliary tract comprise of the diseases of the biliary tract itself (e.g., bacterial cholecystitis, cholangitis, cholangiohepatitis, and necrotizing cholecystitis) and extrahepatic bile duct obstruction (EBDO) in canines.

#### 3.5.1. Etiology

Bacterial cholecystitis is most commonly caused by aerobic gram-negative bacteria (especially *E. coli, Klebsiella, Pseudomonas*, and* Salmonella* spp) or anaerobes (*Clostridium* spp). Cholelithiasis can predispose to cholecystitis by obstructing the cystic duct, causing gallbladder overdistension and stasis, which enables proliferation of anaerobic organisms [8]. The cause of spontaneous cholelithiasis, although cannot be determined, requires initial nidus formation, retention of particles in the gall bladder, and then sustained growth of the cholelith [9]. The extrahepatic bile duct obstruction may be the result of extraluminal compressive causes (e.g., neoplasia, stricture, diaphragmatic hernia, and congenital anomalies of the extrahepatic biliary tract) and intraluminal obstructive causes (e.g., cholelithiasis, inspissated bile, and liver flukes) [27]. Cholangiohepatitis and cholecystitis is most commonly associated with ascending infection [1]. Cholecystitis as generalized gall bladder wall thickening is associated with acute pyelonephritis, portal hypertension, chronic renal diseases, and hepatitis [24]. Biliary sludge which is regarded as a precursor of cholecystolithiasis in humans is frequently identified as an incidental finding in canines, and its cause and clinical significance are not fully understood [28, 29].

#### 3.5.2. Clinical Manifestations

In cholecystitis, the clinical signs include anorexia, lethargy, fever, abdominal pain, vomiting, diarrhoea, and septic shock due to septic bile peritonitis following acute rupture of gall bladder. Dogs with cholelithiasis are often asymptomatic and clinical signs (jaundice, anorexia, weight loss, vomiting, and dehydration) are most likely when cholelithiasis is complicated by bacterial infection, EBDO, perforation of the gall bladder or bile ducts [8]. Regardless of the underlying disorder, typical clinical signs with biliary obstruction are jaundice, acute or chronic vomiting, anorexia, depression, weight loss, diarrhoea, acholic faeces, excessive bleeding, and occasionally vague cranial abdominal pain [9]. Physical examination findings may show fever, cranial abdominal discomfort, jaundice, hepatomegaly due to bile engorgement, abdominal distension, and shock. Excessive bleeding and acholic faeces are indicative of chronic and complete common bile duct obstruction [9].

#### 3.5.3. Laboratory Evaluation

On the CBC, a mild neutrophilia and mild nonregenerative anaemia are common. Neutrophilia with left shift suggests the possibility of acute pancreatitis or abscess, bacterial cholangitis/cholecystitis, or biliary rupture [8, 9]. Serum chemistry findings reveal markedly increased activity of ALP and GGT, cholesterol, bile acids, and bilirubin. Urinalysis findings reveal bilirubinuria and absence of urobilinogen. With vitamin K malabsorption, findings include prolonged PT, APTT, and activated clotting time.

#### 3.5.4. Radiographic Features

Radiographic findings depend on the underlying cause of obstruction and may include cholelithiasis with radiopaque densities in the area of gall bladder or bile duct, emphysematous cholecystitis, pancreatitis, and mass lesion [8]. Microhepatica may be appreciated radiographically with chronic biliary obstruction in dog leads to biliary cirrhosis [8, 9]. There may be complete loss of abdominal detail with septic bile peritonitis [9]. Choleliths may be radiographically recognized as discrete rounded radiopacities in the cranial right ventral shadow.

#### 3.5.5. Ultrasonographic Features

**Figure 24:**
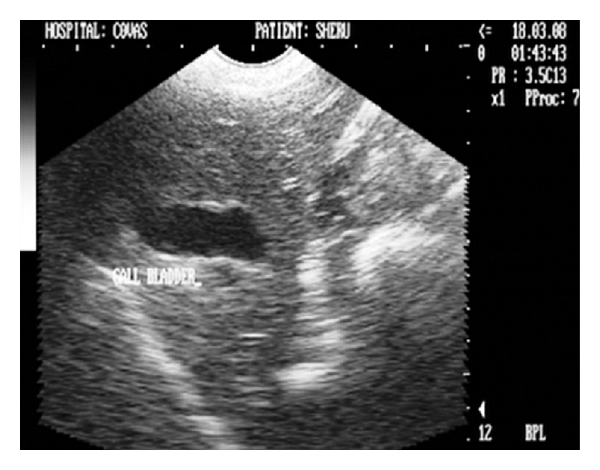
Hepatic sonogram (2D) in sagittal scan of a five-year-old intact Pomeranian male dog depicting increased parenchymal echogenicity with hyperechoic double rim appearance of GB wall.

**Figure 25:**
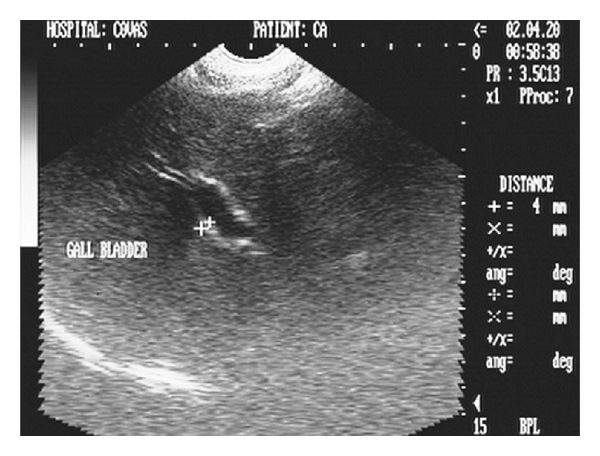
Two-dimensional ultrasonographic appearance in sagittal scan of cirrhosed liver lobe in a seven-year-old intact mixed-breed dog depicting collapsed and irregularly shaped gall bladder with echogenic and thickened (4 mm) GB wall.

The gall bladder wall, owing to cholecystitis gives thickened appearance due to visualization of both the inner and outer walls and is perceived as double rim effect [30]. Double rim effect of gall bladder wall in cholecystitis has also been reported [26]. A hyperechoic thickened GB wall with echogenic bile and biliary sludge were the features associated with cholecystitis [31]. Cholecystitis is sonographically characterized by a hyperechoic gall bladder wall with thickness more or equal to 3 mm [32] and may have thickness of more than 4 mm in cases of neoplasia [33] (Figures 24 and 25). In cholecystitis of inflammatory origin, the thickened gall bladder wall may be isoechogenic with hepatic parenchyma [33].

**Figure 26:**
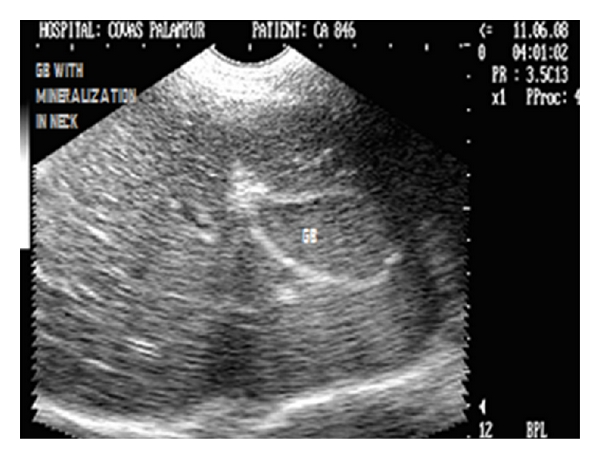
Longitudinal sonogram (2D) of liver of two and half-year-old, intact female Pointer dog showing cholecystitis as echogenic thickened GB wall and mineralization in the neck of gall bladder with acoustic shadowing along with hyperechoic parenchyma.

The choleliths have been observed as echogenic/hyperechoic foci within the dependent lumen of the gall bladder, with some degree of acoustic shadowing [16, 34] (Figure 26).

#### 3.5.6. Histopathological Features

Histopathologic changes in the gall bladder, bile ducts, and liver may be absent with uncomplicated cholelithiasis. However, mild cholangitis and cholecystitis are common [8].

### 3.6. Hepatobiliary Neoplasia

#### 3.6.1. Etiology

The cause of naturally occurring primary hepatic neoplasms in dog is unknown. The possible causes of focal hepatic enlargement include cysts, granulomas, abscesses, neoplasia, regenerative nodules, haematomas, and rarely liver lobe torsion [7]. Noncystic cavitary lesions have been described as mixed areas of anechoic, hypoechoic and hyperechoic patterns resulting from neoplasia [35]. These noncystic cavitary lesions may also result from acquired collections of blood, necrotic fluid, or cellular debris contained within hematomas, nodular hyperplasia, or large neoplastic masses [36]. Invasiveness of hepatocellular carcinoma and cholangiocellular carcinoma with their radiographic appreciation as hepatomegaly has been reported [7], whereas inflammatory diseases or neoplasia, hepatic venous congestion, fat infiltration, cholestasis, cirrhosis, infiltrative diseases (amyloidosis or lipidosis), and storage diseases have been described as the potential causes of diffuse hepatomegaly [11]. Hepatic neoplasia can lead to symmetric hepatomegaly in 25% of the affected dogs [37].

#### 3.6.2. Clinical Manifestations

**Figure 27:**
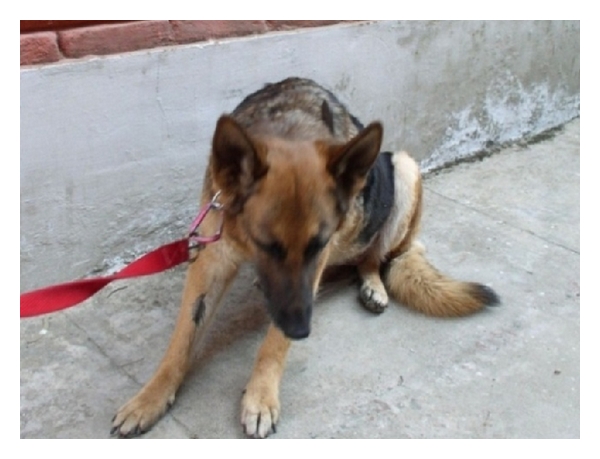
Cranial abdominal discomfort exhibited by a nine-year-old spayed female German Shepherd Dog with hepatic and splenic mass by assuming “position of relief.”

The most consistent signs are anorexia, lethargy, weight loss, vomiting, abdominal distension, polyuria/polydipsia, jaundice, diarrhoea, and excessive bleeding. The potential findings in hepatobiliary neoplasia may include a cranial abdominal mass and hepatomegaly with hepatodynia, pallor, jaundice, and cachexia [8, 9] (Figure 27).

#### 3.6.3. Laboratory Evaluation

Potential haematologic findings with hepatic neoplasia include anaemia (nonregenerative/regenerative) and leucocytosis, whereas biochemical findings may be variable and nonspecific for liver disease [9]. Increases in ALT and ALP usually are mild to marked but may be normal, especially with metastatic tumours [8]. Other findings include hyperbilirubinemia, hypoalbuminaemia, hyperglobulinaemia, increased SBA concentration, prolonged BSP dye retention, and abnormal coagulation tests [9].

#### 3.6.4. Radiographic Features

**Figure 28:**
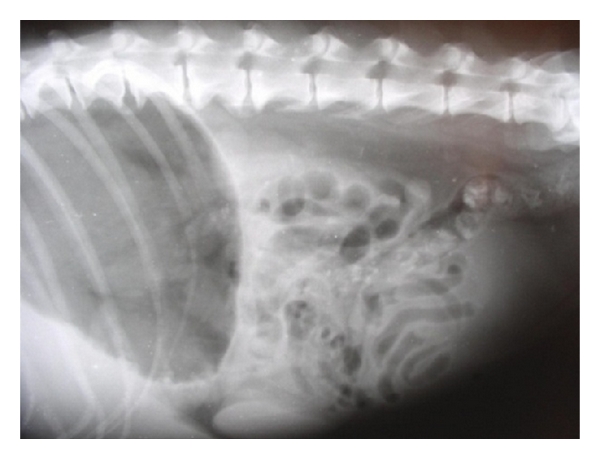
Right lateral radiograph of a nine-year-old spayed female, German shepherd dog revealing hepatic nodular masses on the caudal visceral margins of right liver lobes irregular. Visualization was enhanced by the negative contrast provided by air in gastric fundus.

The irregular hepatic silhouette has been documented as a radiographic feature of nodular hyperplasia [7], whereas focal hepatic enlargement can be detected by bulge or alteration in the hepatic margin or the localized displacement of the fundus, gastric body, pylorus, right kidney, cranial duodenal flexure, transverse colon, and head of the spleen or diaphragm [16]. Liver lobe enlargement displaces the body and pyloric regions of the stomach dorsally and to the left [38] (Figure 28).

**Figure 29:**
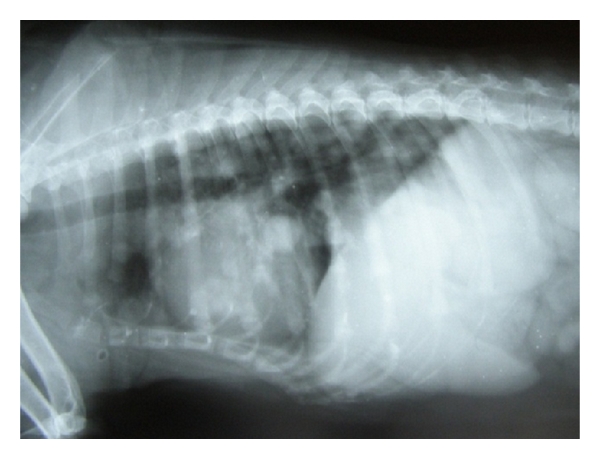
Lateral abdominal radiograph of an 8-year-old intact male, mixed-breed dog with cutaneous tumour and pulmonary metastasis demonstrating generalized hepatomegaly.

Severe diffuse hepatomegaly can be easily demonstrated on lateral radiographic projections as substantial portion of the caudal liver margin projecting beyond the costal arch along with rounding of the caudal liver edges. It may also be evidenced by caudodorsal displacement of stomach in lateral projection and caudally towards the left in ventrodorsal projections [7]. Hepatomegaly can be radiographically evidenced as increased distance between the stomach and diaphragm or caudal displacement and ventral covering of the right kidney by the liver [39]. Hepatomegaly is reliable radiographic sign of liver disease which may be diffuse, with uniform enlargement of all lobes or focal, with enlargement of only a single lobe [11] (Figure 29).

#### 3.6.5. Sonographic Features

**Figure 30:**
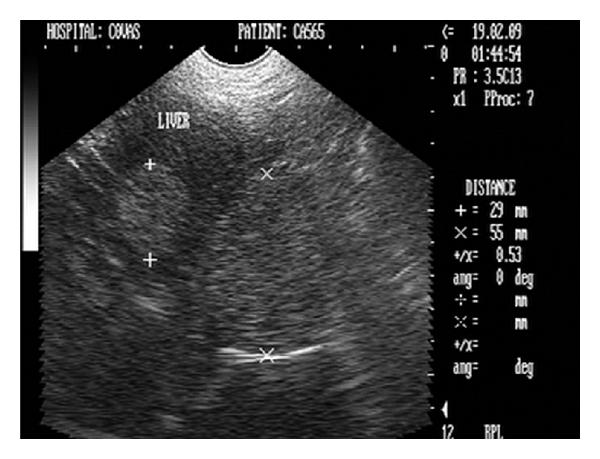
Transverse ultrasonogram (2D) of liver in a nine-year-old spayed female, German shepherd dog revealing two hyperechoic homogenous circular nodular masses measuring 55 mm and 29 mm.

**Figure 31:**
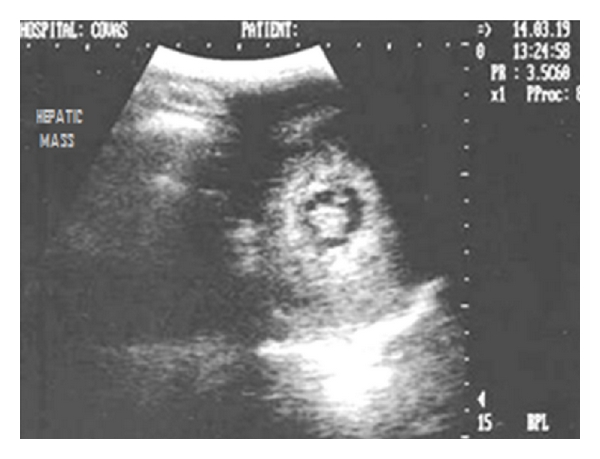
Sagittal sonogram (2D) of liver in a ten-year-old intact Labrador retriever dog affected by a perianal tumour showing hyperechoic hepatic mass with central lytic changes.

**Figure 32:**
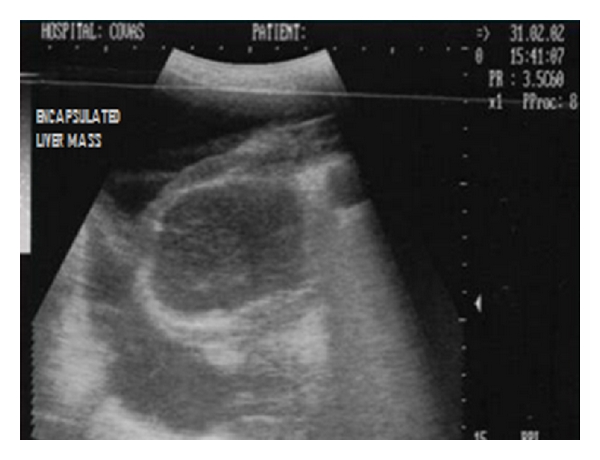
Two-dimensional ultrasonographic transverse scan in a five-year-old intact mixed-breed dog showing an encapsulated hypoechoic hepatic mass with irregular margins.

Focal nodular hyperplasia lesions have been documented as well defined solid masses having a variable, often greater echogenicity than the normal liver parenchyma, and a central highly echogenic area. Hepatic lymphosarcoma may be sonographically characterized as a diffuse, decreased or increased echogenicity in a normal to enlarged liver [40]. Hepatic lymphosarcoma may also be observed as multifocal poorly circumscribed hypoechoic areas or well-circumscribed hypoechoic nodules surrounded by areas of hyperechogenicity (target lesions) [41]. Hepatic lymphoma has been sonographically characterized by decreased hepatic echogenicity in canines [22], whereas a solitary hyperechoic mass has been described as the most common appearance of canine hepatocellular carcinoma [42]. Primary hepatic neoplasia has a variable sonographic appearance ranging from a very large, moderately circumscribed, infiltrating mass bulging beyond the normal liver margins with an echogenicity slightly more mixed than normal liver [43]. Ultrasonography is sensitive in the detection of small focal lesions, although lesions less than 2 cm are hard to identify with this imaging modality. It is also not so useful if the lesion is less than 2 cm in diameter and isoechoic with normal tissue [24] (Figures 30, 31, and 32).

**Figure 33:**
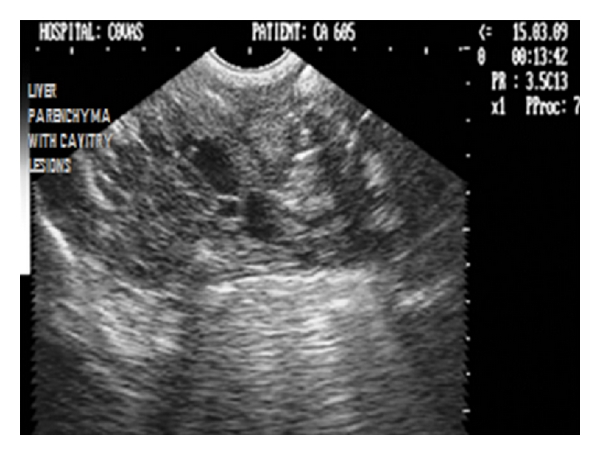
Sagittal sonograph (2D) of liver with extensive noncystic cavitary lesions in hepatic parenchyma in a nine-year-old intact male Boxer dog.

**Figure 34:**
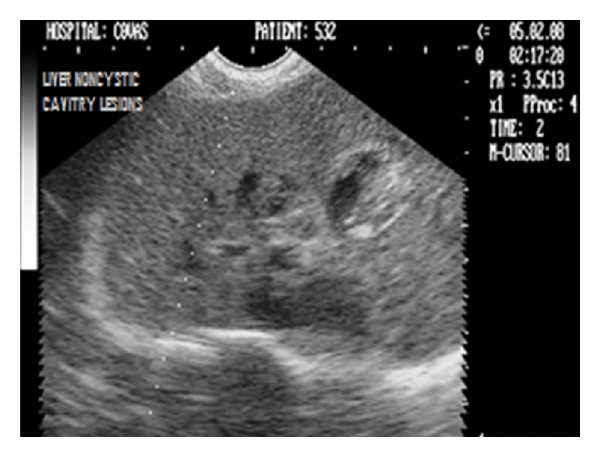
Two-dimensional ultrasonographic sagittal scan of right liver lobe in seven-year-old mixed-breed intact male dog showing noncystic cavitary lesions with generalized increase in parenchymal echogenicity.

**Figure 35:**
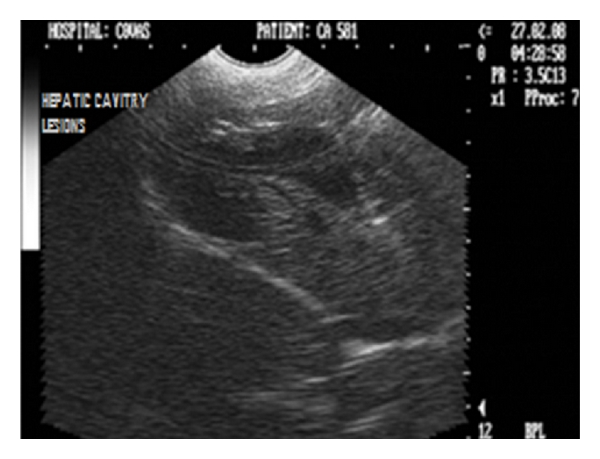
Ultrasonogram (2D) of liver in longitudinal scan revealing large poorly echogenic area with internal septations in a one and half-year-old neutered male Doberman pinscher dog.

Noncystic cavitary lesions (e.g., abscesses, hematomas, hepatic nodular hyperplasia, and cavitated neoplasms) have been documented as mixed or complex echoic structures with either well- or poorly defined borders [36]. Irregular, poorly defined borders in such lesions result in edge shadowing that is less apparent than that occurring from well-defined cysts (Figures 33, 34, and 35).

#### 3.6.6. Laparoscopic Features

**Figure 36:**
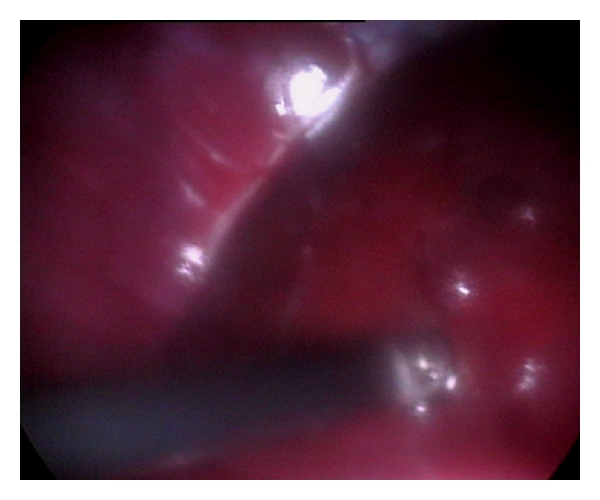
Laparoscopic direct visualization of liver in 5-year-old mixed-breed male dog showing a nodular hyperplasia which remained undetected ultrasonographically.

Laparoscopy provides advantage of direct visualization and extracting valuable information regarding the conditions of the tissues and the extension of the lesions while visualizing hepatic tumours, hepatic necrosis, splenic and pancreatic atrophy, hepatofrenic adhesion, intestinal neoplasia, and piometritis [44]. The diagnostic precision of laparoscopy is tremendous, and it can easily visualize metastatic lesions/masses as small as 0.5 cm or less, which are not observed easily by other diagnostic techniques [3] (Figure 36).

#### 3.6.7. Histopathologic Features

The most common types of primary hepatic cancer reported in male dogs are hepatocellular carcinoma and intrahepatic cholangiocellular (bile duct) carcinoma in female dogs. Hepatocellular adenoma are observed less frequently in either gender [9]. Metastases to the liver by hematogenous routes from distant sites are much more common, arising from gastrointestinal, pancreatic and mammary adenocarcinoma and from hemangiosarcoma [9]. Liver involvement is also common in canines with lymphoma.

## 4. Conclusion

As the liver is physiologically and anatomically diverse, there is no single test that adequately identifies hepatic disease or its underlying cause. For this reason, a battery of tests must be used to diagnose the hepatobiliary affections. A reasonable package of screening tests recommended for an animal suspected of having hepatobiliary disease includes a complete blood count (CBC), serum biochemical profile, urinalysis, faecal analysis, survey radiography, and ultrasonography. The primary indication so obtained may suggest evidence of hepatobiliary disease that can be confirmed by other, more specific tests including advanced diagnostic imaging procedures. The need for other laboratory tests, that is, abdominocentesis, coagulation profile, and evaluation of fasting plasma ammonia concentration, and so forth is determined by each animal's history and physical examination findings. A definitive diagnosis of hepatobiliary affection should be confirmed by hepatic biopsy and histopathological examination.
